# SMAC Mimetic BV6 Co-Treatment Downregulates the Factors Involved in Resistance and Relapse of Cancer: IAPs and Autophagy

**DOI:** 10.3390/biology11111581

**Published:** 2022-10-27

**Authors:** Sahar Rafat, Prabhakar Singh, Kamlesh Kumar Pandey, Saleh A. Almatroodi, Mohammed A. Alsahli, Ahmad Almatroudi, Arshad Husain Rahmani, Kapil Dev

**Affiliations:** 1 Department of Biotechnology, Jamia Millia Islamia, New Delhi 110025, India; 2 Department of Anatomy, All India Institute of Medical Sciences, New Delhi 110025, India; 3 Department of Medical Laboratories, College of Applied Medical Sciences, Qassim University, Buraidah 51452, Saudi Arabia

**Keywords:** autophagy, apoptosis, IAPs, smac mimetics, breast cancer, TRAIL, TNFα

## Abstract

**Simple Summary:**

Breast cancer is the most prominent cancer among women. Therapeutic resistance and reoccurrence of cancer after various therapies, such as chemotherapy, radiotherapy etc., is the major cause of death. In various studies, it has been shown that dysregulation or defect in the cell death mechanisms such as apoptosis and autophagy is the key reason behind the issues. An increase in the level of inhibitors of apoptosis (IAPs) proteins and autophagy is founded in various cancers. Besides, the SMAC proteins that inhibit the action of IAPs are also found to be downregulated. In this study, BV6, a SMAC mimetic compound, not only induces apoptosis via inhibiting the action of IAPs but also downregulates autophagy. In addition, promising anticancer results were obtained after treating the breast cancer cells with BV6 in combination with death ligands such as TNF-related apoptosis-inducing ligand (TRAIL) and Tumor necrosis factor-α (TNF-α). It is an investigational study, and to the best of our knowledge, our study, for the first time, demonstrated the effect of BV6 on autophagy. This study presents the significance of SMAC mimetic compounds in the downregulation of factors responsible for cancer resistance and reoccurrence. The study may well provide a potent therapeutic target for the development of novel anti-cancer therapy using SMAC mimetics.

**Abstract:**

Cancer is the utmost common disease-causing death worldwide, characterized by uncontrollable cell division with the potential of metastasis. Overexpression of the Inhibitors of Apoptosis proteins (IAPs) and autophagy correlates with tumorigenesis, therapeutic resistance, and reoccurrence after anticancer therapies. This study illuminates the role and efficacy of smac mimetic compound BV6 alone and in co-treatment with death ligands such as TRAIL and TNFα in the regulation of cell death mechanisms, i.e., apoptosis and autophagy. In this study, MTT assays, wound healing assays, and cellular and nuclear morphological studies were done. DAPI staining, AO/EtBr staining and AnnexinV/PI FACS was done to study the apoptosis. The expression of IAPs and autophagy biomarkers was analyzed using Real time-PCR and western blotting. Meanwhile, TEM demonstrated autophagy and cellular autophagic vacuoles in response to the BV6. The result shows a promising anti-cancer effect of BV6 alone as well as in combinational treatment with TRAIL and TNFα, compared to the lone treatment of TRAIL and TNFα in both breast cancer cell lines. The smac mimetic compound might provide an alternative combinational therapy with conventional anticancer therapies to tackle their inefficiency at the advanced stage of cancer, cancer resistance, and reoccurrence. Also, IAPs and autophagic proteins could act as potent target molecules for the development of novel anti-cancer drugs in pathogenesis and the betterment of regimens for cancer.

## 1. Introduction

Breast cancer is the most prominent and rampant cancer among women surpassing lung cancer [1]. Despite various improvements in breast cancer regimens, none of the treatment options is efficient for remission at the advanced stages of the disease [2]. The incidence rate and mortality rate of breast cancer account for 24.5% and 15.5%, respectively [3]. Therapeutic resistance and reoccurrence developed after chemotherapy is the major causes of cancer-related deaths. The noteworthy reason is the failure of the cell death mechanism by which they escape from death-inducing drugs and metastasize [4]. Apoptosis is a well-known type I programmed cell death mechanism which is highly regulated in normal cells [5]. One of the regulatory proteins is the Inhibitor of apoptosis protein (IAPs), a class of proteins that inhibit apoptosis via their baculoviral IAP repeat (BIR) domains and ubiquitinate the caspase proteins. They are overexpressed in various cancer and are involved in hindering cancer cells from undergoing apoptosis and developing resistance to anticancer therapies [6,7]. Endogenous inhibitors of IAPs, known as Second mitochondria-derived activator of caspase (SMAC) or Direct IAP-Binding protein with Low PI (DIABLO) proteins, are released from the mitochondria to cytosol. These proteins bind to the IAPs that activate caspase protein action and cell apoptosis [2,8,9]. Various small molecules or compounds mimicking SMAC proteins activity (Smac mimetics) demonstrated a promising role in sensitizing cancer cells to cytotoxic drugs and are under clinical trial [10,11,12]. In a recent study, a Smac mimetic compound MV1 was conjugated to a ligand of Fn14, and TWEAK suppressed squamous cell carcinoma (SCC) both in vitro and in vivo [13].

The development of tumour resistance against various chemotherapy drugs is the major obstacle that ultimately leads to the reoccurrence of cancer. The most common and promising approach is stimulating the death receptors and the use of death ligands such as TRAIL and TNFα to induce apoptosis in cancer cells. However, various cancer types adapt and develop resistance against these death ligands. Apart from apoptosis, the type II cell death mechanism known as autophagy is also an evolutionary conserved catabolic process. Difficulty in the autophagy mechanism could lead to the rise of various pathological conditions, including cancer. In the cancer context, autophagy acting a paradoxical role. It provides cancer cell resistance and maintains survival in response to therapies [14,15,16,17,18].

In this study, we have gaged the anticancer activity of BV6 [N, N′-(hexane-1,6-diyl) bis (1 {(2S)-2-cyclohexyl-2-[(N-methyl-L-alanyl)-amino]-acetyl}-L-prolyl-beta-phenyl-L phenylalaninamide)] on breast cancer cell lines i.e., MCF7 and MDA-MB-231. To the best of our knowledge, for the first time, the potential effect of BV6 on autophagy was studied. Also, for the first time, the combined effect of SMAC mimetic BV6 and death ligands TRAIL and TNFα on autophagy was investigated. Our study may provide an alternate combination therapy or the development of novel anti-cancer therapy using SMAC mimetic compounds to tackle the major issues.

## 2. Materials and Methods

### 2.1. Cell Culture and Maintenance

Cancer cells MCF-7 and MDA-MB-231 were purchased (National Centre for Cell Science (NCCS), Pune, India) and maintained in Dulbecco’s modified Eagle’s medium (DMEM) with 10% (*v*/*v*) heat-inactivated FBS (Gibco, Brazil), 1% penicillin/streptomycin (Gibco, Thermo-fisher, Waltham, MA, USA), under sterile incubator with humidified CO_2_ at 5%, and temperature 37 °C. Cells were passaged using 0.05% Trypsin (Gibco, Stratford, ON, Canada) routinely after 2–3 days.

### 2.2. MTT Assay

MCF-7 and MDA-MB-231 breast cancer cells (8000 cells/well) for 24 **h** before the treatment of the drug were cultured on 96-well microtiter plates. Different concentrations of BV6 (Millipore Corp., Burlington, MA, USA), Recombinant human TRAIL (GF092, Millipore, Burlington, MA, USA) and Recombinant human TNFα (GF023, Millipore, Burlington, MA, USA) alone and in combinations were treated for 24 h. After washing with PBS, 20 μL of MTT (Himedia, Thane, India) per well for 3 h at 37 °C was incubated, followed by the addition of 100 µL of DMSO to each well. After half an hour, a plate was placed on a microtiter plate reader (Bio-Rad microplate reader, Bio-rad, Hercules, CA, USA) and tested at a wavelength of 570 nm. Cell viability (%) was calculated as (absorbance of test sample/absorbance of control) 100% and IC50 with the help of Graphpad Prism software.

### 2.3. Cellular Morphology

Breast cancer cells MCF-7 and MDA-MB-231 are grown on 24-well flat-bottom microtiter plates (Costar 3524, Corning Inc., Corning, NY, USA), and were treated with different concentrations of BV6 (0.5 μM, 1.0 μM), TRAIL (50 ng/mL) and TNFα (50 ng/mL) alone and in combination. After 24 h, the morphology of MCF-7 and MDA-MB-231 cells were observed under an inverted phase-contrast microscope (Olympus, Tokyo, Japan).

### 2.4. Wound Healing

To measure the 2D migration of cancer cells, a wound/scratch was created with the help of a 10 µL sterile micropipette tip on a 100% confluent cell monolayer seeded in 12-well plates. Media was removed, cells gently washed twice with PBS, and culture media containing BV6 was added into each well. Images were taken at 0 h and after 24 h of incubation with and without compound.

### 2.5. Acridine Orange and Ethidium Bromide Staining

Acridine Orange and Ethidium Bromide (AO/EtBr) are used for staining live cells, apoptotic cells and necrotic cells. After more than 80% confluency of cells seeded in a 12-well plate was treated. These cells were trypsinized and centrifuged at 1000 rpm for 5 min. Cells were washed with PBS and resuspended in AO/EtBr solution (1:1). After the incubation of 15 min, the cell suspension was placed on a clean glass slide and observed under a fluorescence microscope.

### 2.6. DAPI Staining

Cells were cultured on a glass coverslip in 12 well plates and treated with compounds (for 24 h). After fixing the cells with pre-chilled absolute methanol at −20 °C for 15 min, cells were stained for 5 min with 25 nM DAPI (Invitrogen, Waltham, MA, USA), followed by two times PBS wash. The coverslips were then picked carefully and mounted on a glass slide, and observed under a confocal imaging system from Leica.

### 2.7. Annexin V Staining Assay

Apoptosis quantification was done by Annexin V-FITC/PI (propidium iodide) co-staining assay (BD Biosciences) using the manufacturer’s suggested guidelines. In brief, MCF7 and MDA-MB-231 breast cancer cell lines at 1 × 10^5^ cells/mL were cultured in 24-well plates and treated for 24 h with different concentrations of smac mimetic compound, TRAIL (50 ng/mL) and TNFα (50 ng/mL) alone and in combination. The cells were harvested and centrifuged at 1600 rpm for 5 min. The pellet was resuspended in a 500 μL binding buffer containing 5 μL Annexin V-FITC and PI (50 μg/mL). After 15 min incubation in the dark at room temperature, the cells were analyzed by flow cytometry (BD Aria).

### 2.8. MDC Staining

MCF-7 and MDA-MB-231 cancer cells were seeded and allowed to grow for 24 h. After 50–60% confluency, treatment was done with BV6 (0.5 µM, 1 µM), TRAIL (50 ng/mL) and TNFα (50 ng/mL) alone and in combination. Along with the treated group, control (only medium) and positive control (induced autophagy via starvation) were also maintained. The cells were stained with 0.5 mM Monodansylcadaverine (MDC, Sigma Aldrich, St. Louis, MO, USA) for 30 min at 37 °C. After washing, cells were visualized using a cell imager microscope (ZOE, Bio-rad, Hercules, CA, USA).

### 2.9. Real-Time PCR

RNA (total) was isolated by using TRIzol Reagent (Invitrogen, Waltham, MA, USA). By using the manufacturer’s suggested guidelines of the Verso cDNA Synthesis Kit (Thermo Scientific, Vilnius, Lithuania), 1 μg of RNA was reverse transcribed to form cDNA. Real-time PCR was performed with SYBR green technology on an Applied Biosystems (Foster City, CA, USA) real-time PCR machine. Primers were synthesized by IDT (Coralville, IA, USA) and are as follows: Pair A. XIAP Primers: Sense 5′-GCA CGA GCA GGG TTT CTT TAT ACT GGT G-3′Anti-sense 5′-CTT CTT CAC AAT ACA TGG CAG GGT TCC TC-3′. Pair B. c-IAP1 Primers: Sense 5′-GAA TAC TCC CTG TGA TTA ATG GTG CCGTGG-3′ Anti-sense 5′-TCT CTT GCT TGT AAA GAC GTC TGT GTC TTC-3′. Pair C. c-IAP2 Primers: Sense 5′-GAA TAC TCC CTG TGA TTA ATG CTG CCG TGG-3′ Anti-sense 5′-TCT CTT GCT TGT AAA GAC GTC TGT GTC TTC-3′. Pair D. Survivin Primers: Sense 5′-GCC ATG AAT TCA TGG GTG CCC CGA CGT TGC-3′ Anti-sense 5′-AGC TCT CTA GAG AGG CCT CAA TCC ATG GCA-3′. Pair E. Beclin-1: Sense 5′-AGG ATG ATG TCC ACA GAA AGT GC-3′Anti-sense 5′-AGT GAC CTT CAG TCT TCG GCT G -3′Pair F. LC-3: Sense 5′-AGA CCT TCA AGC AGC GCC G -3′Anti-sense 5′-ACA CTG ACA ATT TCA TCC CG -3′ Pair G. Actin Primers: Sense 5′-CCC CTT CAT TGA CCT CAA CT-3′ Anti-sense 5′-TTG TCA TGG ATG ACC TTG GC-3′. Caspase3 Primers: Sense 5′-TTGTGCCATGCTGAAACAGT-3′ Anti-sense 5′-AGCATGGAAACAATACATGGAA-3′.

### 2.10. Western Immunoblotting

To near 90% confluency breast cancer cell lines, MCF7 and MDA-MB-231 were allowed to grow on cell culture dishes (Corning Costar). After treatment, cells were lysed by RIPA lysis buffer and protease inhibitor (Promega, Madison, WI, USA) and then incubated on ice for 20 min and centrifuged at 16,000 rpm for 20 min. The supernatant was transferred into a new Eppendorf, and the protein concentration was determined by Bradford assay. The 30–50 μg of total protein resolved on SDS/PAGE gel followed by Western blotting. The proteins were transferred from SDS/PAGE gels onto the PVDF membrane (Bio-Rad, Hercules, CA, USA) using a wet transfer system at 75 V from 30 min to 2 h. Blocking is done for an hour with 5% nonfat milk in TBST and specific primary antibodies XIAP (sc-55550), cIAP1 (sc-271419), cIAP2 (sc-517317), MAP LC3a/b (sc-398822), Survivin (sc-17779), Beclin (sc-48341), and Actin (sc-47778, Santa Cruz Biotechnology) probing done overnight at 4 °C. The primary unbound antibodies on membranes were removed and washed thrice with 1X TBST. After proper washing, the membrane was incubated with HRP-conjugated secondary antibodies (sc-516102-CM, Santa Cruz Biotechnology, Dallas, TX, USA) at room temperature for 1 h. Chemiluminescence-based detection ECL substrate (Bio-rad, Hercules, CA, USA) was poured on the membrane and the signals were observed and captured using Chemidoc (Bio-rad, Hercules, CA, USA).

### 2.11. Transmission Electron Microscope (TEM)

TEM is considered a gold standard method to visualize the autophagic vacuoles or the status of autophagy in a cell. Untreated and treated cancer cells were harvested and fixed in a 3% glutaraldehyde solution (Karnovsky’s fixative) at 4 °C overnight. Fixed cells were then treated with 1% osmic acid, followed by step-wise dehydration by gradient ethanol (30–100%) and embedded in an epoxy resin. Using ultramicrotome, samples were cut into ultrathin sections and collected on copper grids. Grids were examined under the electron microscope (CM-10, Philips, Amsterdam, The Netherlands).

### 2.12. Statistical Analysis

All data were analyzed using Graph pad prism version 6.0 statistical software, and the values are expressed as mean ± standard error of the mean (SEM) from three independent experiments. Statistical significance was considered at *p* < 0.05 using Tukey’s multiple comparison test and ANOVA. The asterisks (*) (**) (***) and (****) show *p* < 0.05, *p* < 0.01, *p* < 0.001, and *p* < 0.0001 respectively.

## 3. Results

### 3.1. BV6 Effect on Cell Viability, Cell Morphology and Migration in MCF7 and MDA-MB-231

**Figure 1:**
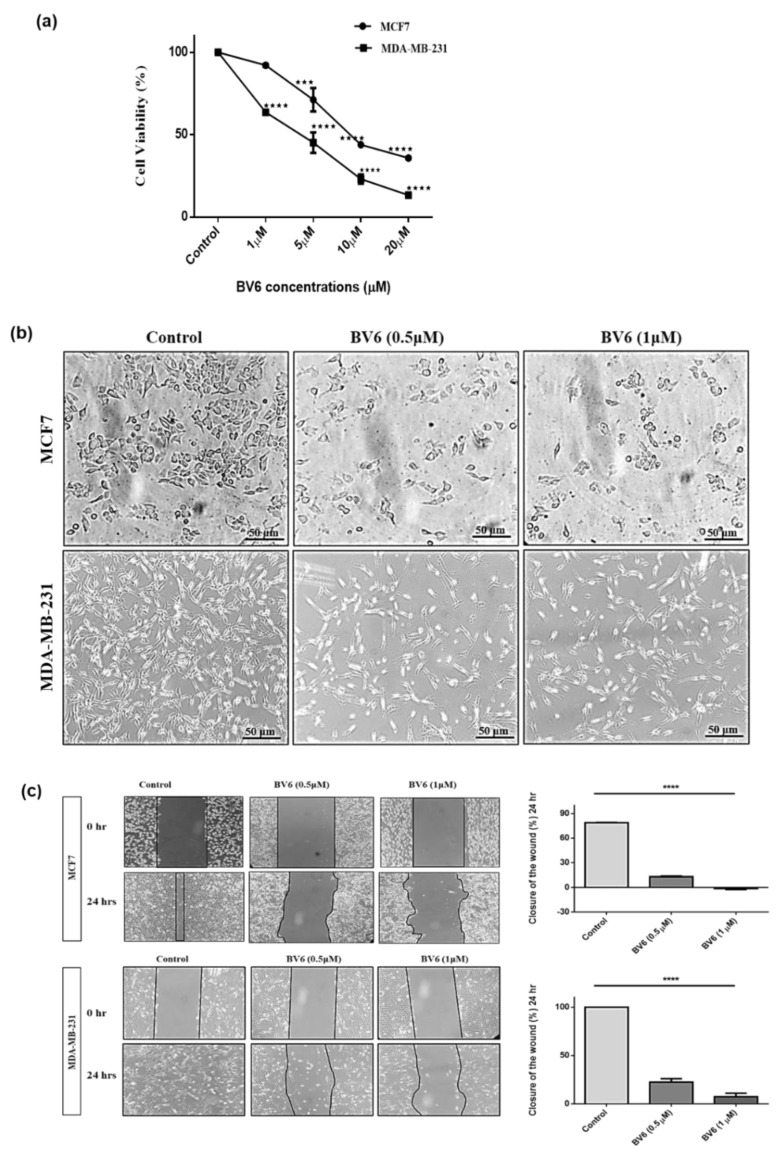
(**a**) BV6 effect on cell viability evaluated by MTT assay: Graph representing that MCF7 and MDA-MB-231 cancer cell viability decreases in a dose-dependent manner. (**b**) Effect of BV6 on MCF7 and MDA-MB-231 cells morphology: untreated cells adhered with normal morphology, whereas treated cells displayed suspended, rounded and shrunken morphology in response to BV6 (40X magnification); scale bar 50 µm. (**c**) Migration by breast cancer cells was assessed by wound/Migration assay with control and 24 h BV6 treated groups. Migration of cells is higher in the untreated group, whereas the migration of cancer cells is impeded by an increase in BV6 concentration. Images of MCF7 and MDA-MB-231 cells captured at 0 hr and after 24 h of scratch assay showing wound closure in response to BV6 compound along with histogram profile demonstrating the percentage closure of the wound by cells/migration of cells after the 24 h of BV6 treatment compared to untreated control cells. Data represented is statistically significant (***) and (****) show *p* < 0.001, and *p* < 0.0001 respectively and is carried out in triplicates displayed as mean ± SEM.

The antitumor activity of BV6 was evaluated by investigating the effect of BV6 on the growth of breast cancer cells MCF7 and MDA-MB-231 using an MTT assay. After 24 h of BV6 treatment, results indicated that BV6 sturdily suppressed the growth and survival of breast cancer. From 30% to 70% decrease in cell viability was observed from 5 μM to 20 μM of BV6 in MCF7. Whereas in the MDA-MB-231 cell line, a 60% decrease in cell viability was seen in response to 1 μM of BV6 to 17% at 20 μM of BV6 (Figure 1a). The IC50 values of BV6 were 5.36 μM and 3.71 μM in MCF7 and MDA-MB-231, respectively.

The change in the morphological characteristics of both cancer cells was observed under inverted phase-contrast microscopy. Untreated/control cells are healthy, showing adhered normal morphology, whereas cells treated with BV6 demonstrated remarkable morphological changes. Cells become round, shrunk, irregular, detached from the surface and were suspended in culture media (Figure 1b).

Cell migration and interaction are studied by wound healing assay. A decrease in wound closure percentage by cells was observed after 24 h of treatment at 0.5 μM and 1 μM of BV6. Wound closure in the control group is 80% and 100% in MCF7 and MDA-MB-231, respectively. The percentage of wound closure after 24 h of 0.5 μM of BV6 treatment is 15% and 22.5% in MCF7 and MDA-MB-231, respectively. Whereas a decrease in closure or increase in wound percentage was observed at a higher dose of BV6, i.e., 1 μM in MCF7 and 7.5% wound closure in MDA-MB-231. Moreover, loss in cell number and its cell-cell interaction is observed in MDA-MB-231 cells in response to BV6 (Figure 1c).

### 3.2. BV6 Induces Apoptosis in MCF7 and MDA-MB-231

**Figure 2:**
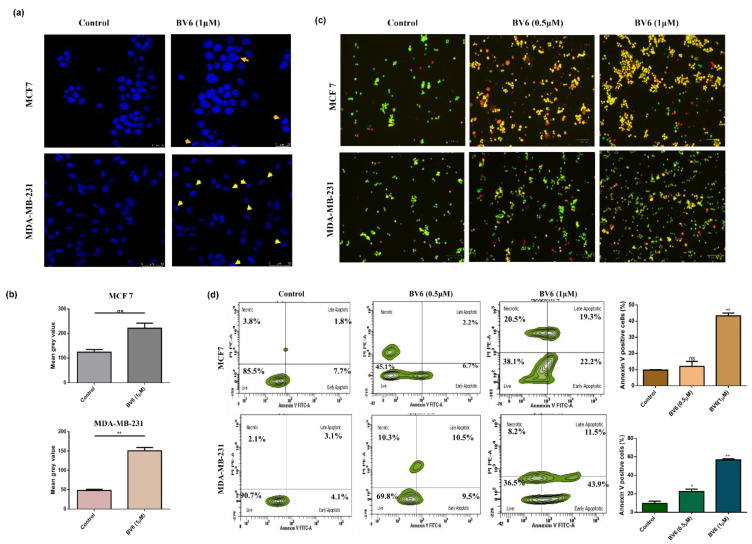
(**a**) DAPI staining of MCF7 and MDA-MB-231 breast cancer cells: Treatment of BV6 for 24 h shows apoptosis induction. DAPI staining images demonstrating changes in the nuclear morphology (Yellow arrows) like blebbing, condensation of chromatin, nuclear shrinkage and apoptotic bodies formation in response to smac mimetic compound BV6. (**b**) Quantitation of the DAPI fluorescence signal as the mean grey value in cells. (**c**) AO/EtBr (Acridine Orange/Ethidium Bromide) staining in MCF7 and MDA-MB-231 breast cancer cells treated with BV6 for 24 h illustrating live cells, early apoptotic cells, late apoptotic cells and necrotic cells in green, orange, yellow and red fluorescence respectively. (**d**) Flow cytometry analysis Annexin V/PI staining to determine the apoptosis: Quadrants showing the proportion of live cells, early apoptotic cells, late apoptotic cells, and necrosis in control and BV6 (0.5 μM and 1 μM) treated cells. Histogram profile demonstrating the percentage of Annexin V positive cells, i.e., apoptosis induced by BV6. Data represented is statistically significant, (*) and (**) show *p* < 0.05 and *p* < 0.01, respectively, and is carried out in triplicates displayed as mean ± SEM.

Apoptotic body formation, blebbing, chromatin condensation and fragmentation are the characteristic features of apoptosis. Nuclear morphology is studied by DAPI staining using confocal microscopy. Control cell lines displayed normal and intact nuclear morphology, whereas BV6-treated cells for 24 h showed changes in the morphology of the nucleus (yellow arrows in Figure 2a). Furthermore, apoptosis and cell death were elucidated by AO/EtBr (Acridine Orange/Ethidium Bromide) staining using fluorescence microscopy. BV6 induces apoptosis, illustrated by the increase in orange and yellow fluorescence, along with cell death shown in red fluorescence. Cells showing green fluorescence indicating viable cells. A higher number of cells with green fluorescence was observed in the untreated control group (Figure 2b). Hence proving that the drug exhibits apoptotic potential on both breast cancer cell lines in a dose-dependent manner.

**Figure 3:**
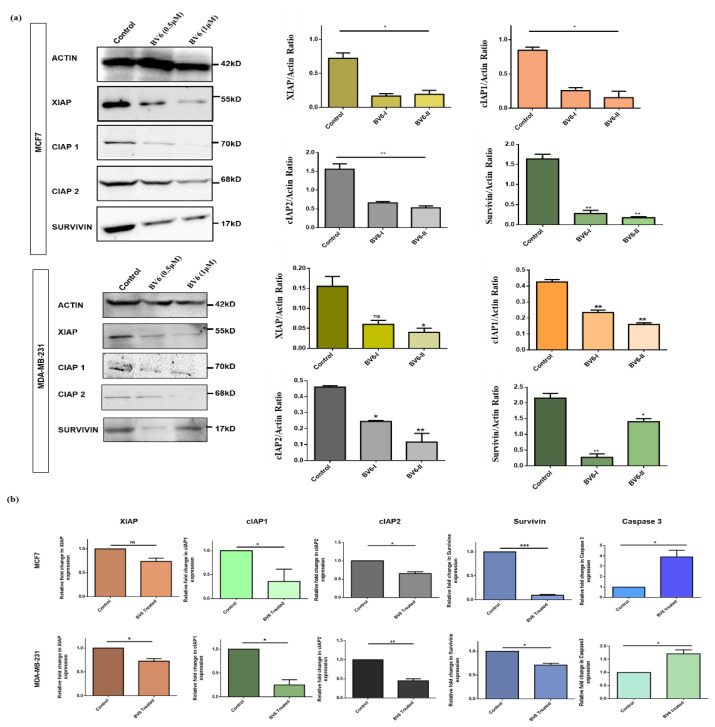
Relative protein and mRNA expression of IAPs in untreated and BV6 treated MCF7 and MDA-MB-231cells determined by Real-time PCR and western blotting, respectively. (**a**) Western Blot images showing the protein bands of untreated control and treated breast cancer cells with 0.5 μM and 1 μM of BV6. Histogram displaying the fold change in the XIAP cIAP1, cIAP2 and survivin protein expression levels normalized by Actin. (**b**) Fold change in XIAP, cIAP1, cIAP2, survivin and caspase3 mRNA expression levels normalized by Actin in both breast cancer cell lines. The experiments are carried out in triplicates displayed as mean ± SEM, and the data represented is statistically significant (*), (**), and (***) show *p* < 0.05, *p* < 0.01 and *p* < 0.001, respectively.

Flow cytometry analysis was done to quantify the possible induction of apoptosis using Annexin V/PI staining. Cell populations that are AnnexinV positive are apoptotic cells. AnnexinV+/PI− are early apoptotic cells, whereas AnnexinV+/PI+ are late apoptotic. AnnnexinV-/PI+ stained cells are dead cells, while AnnexinV−/PI− are live cells. In MCF7, the apoptosis proportion of annexin-positive cells is increased approximately from 5% at 0.5 μM of BV6 to 30% at 1 μM of BV6 compared to the control group (10%). In MDA-MB-231 breast cancer cells, an increase in apoptosis is observed from 9.5% of annexin positive cell population in the control group to 22.5% of annexin positive cell population at 0.5 μM of BV6 and 56.7% of annexin positive cells at 1 μM of BV6 (Figure 2c). Moreover, the apoptosis induction is substantiated by an increase in the level of cleaved caspase 3 via real-time PCR (Figure 3b).

### 3.3. BV6 Exposure Downregulated the Expression of IAPs

The expression of IAPs in response to BV6 is demonstrated by real-time PCR and western blotting. Both cell lines, in response to BV6 in a dose-dependent manner, showed downregulation of XIAP, cIAP1, cIAP2 and survivin protein expression compared to the untreated control group in western blotting analysis (Figure 3a). Consistent results of downregulation of XIAP, cIAP1, cIAP2 and survivin RNA expression levels treated with BV6 smac mimetic compound compared to control are demonstrated by real-time PCR (Figure 3b).

### 3.4. Inhibition of Autophagy in MCF7 and MDA-MB-231 Cells Treated with BV6

**Figure 4:**
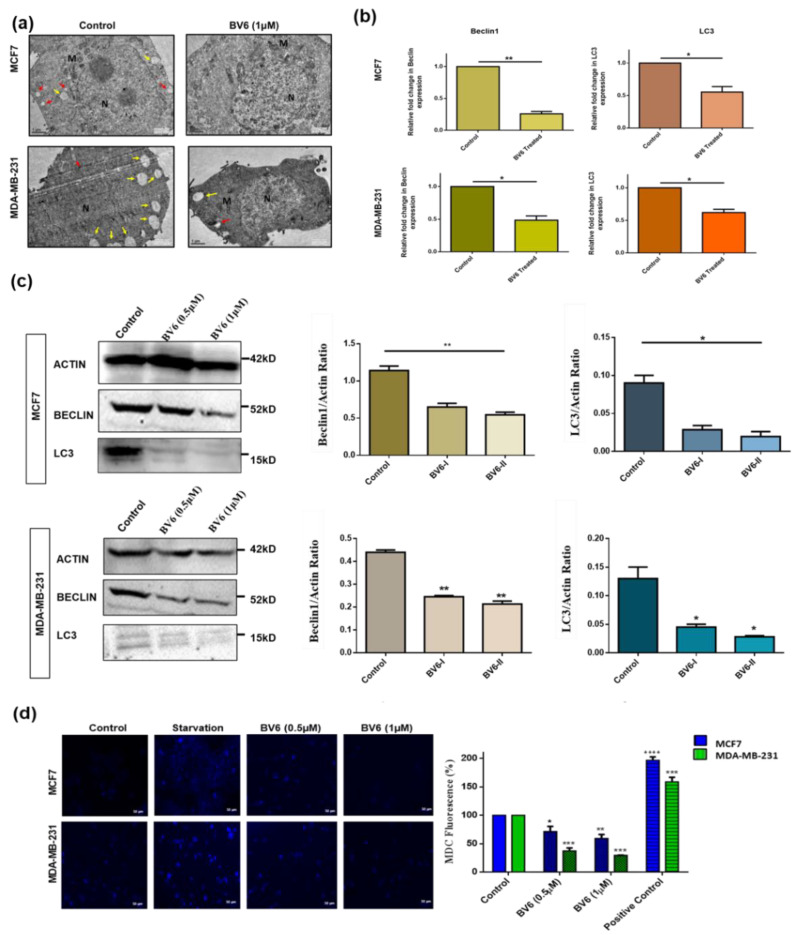
Autophagy in MCF7 and MDA-MB-231 cells treated with BV6: (**a**) TEM images show a normal double-membrane nucleus surrounded with normal-appearing mitochondria along with accumulated autophagic vacuoles such as autophagosomes and autolysosomes. M represents mitochondria and N represents the nucleus. (**b**) The relative fold change in Beclin-1 and LC3 mRNA expression levels normalized by Actin in both breast cancer cell lines. (**c**) Western Blot images showing the protein bands of untreated control and treated breast cancer cells with 0.5 μM and 1 μM of BV6. Histogram displaying the change in the Beclin-1 and LC3 protein expression levels normalized by Actin. (**d**) Fluorescence images captured from fluorescence microscopy of MDC staining showing autophagic vacuoles or autophagosome accumulation in untreated (control), starved cells (Positive control) and BV6 treated cells. Histogram profile representing the mean fluorescence intensity of BV6 treated MCF7 and MDA-MB-231 compared to the fluorescent intensity of untreated control and positive control. BV6-I represents BV6 (0.5 μM), and BV6-II represents BV6 (1 μM) in the histogram profile of Western blot. The experiments are carried out in triplicates displayed as mean ± SEM, and the data represented is statistically significant, (*), (**), (***), and (****) show *p* < 0.05, *p* < 0.01, *p* < 0.001, and *p* < 0.0001 respectively.

Since the electron microscope is regarded as the gold standard in monitoring autophagy, we qualitatively assessed the autophagy via Transmission Electron Microscopy (TEM). Untreated breast cancer cell lines MCF7 and MDA-MB-231 show normal double-membrane nuclei surrounded with normal-appearing mitochondria along with accumulated autophagic vacuoles such as autophagosomes and autolysosomes. Autophagosomes (Red arrows in Figure 4a) are characterized by the vacuole-like structure of bilayer membranes containing cytoplasmic components such as organelles, whereas autolysosomes (Yellow arrows in Figure 4a) appear in the single membrane or fusion state with autophagosomes. In treatment with BV6 for 24 h, the number of autophagic vacuoles decreases (Figure 4a), suggesting that BV6 inhibits the autophagy process in MCF7 and MDA-MB-231 breast cancer cells. Autophagy downregulation is further supported by the evaluation of the expression of autophagic markers Beclin1 and LC3 in BV6-treated MCF7 and MDA-MB-231 cancer cell lines with the help of real-time PCR. 0.75-fold and 0.5-fold decrease in Beclin and LC3 expression in BV6 treated MCF7 cell line is observed, whereas 0.45-fold and 0.38-fold decreases in the expression of beclin and LC3 in MDA-MB-231-treated cells compared to the untreated group (Figure 4b).

Next, we investigated the change in the protein expression of Beclin1 and LC3 in treated cells in comparison to control by western blotting. Downregulation of beclin1 and LC3 protein expression is observed in response to BV6 in both MCF7 and MDA-MB-231 breast cancer cell lines (Figure 4c). Autophagy in breast cancer cell lines is also demonstrated by MDC staining. MDC is an autofluorescent marker that accumulates in autophagic vacuoles or autophagosomes, especially with phosphatidylethanolamine (PE) present in the autophagosome membrane. Concentration-dependent inhibition of stained autophagic vacuoles observed in BV6 treated cells. Autophagic vacuoles are marked rises in both starved breast cancer cell lines (Positive control) compared to untreated control. The histogram profile shows a 30–50% decrease in mean fluorescence intensity of BV6 treated MCF7 and a 70–75% decrease in mean fluorescent intensity in the MDA-MB-231 treated cell line compared to the fluorescent intensity of untreated control of both cell lines. In comparison to the control group, starved cells show about a 96%-fold increase in MCF7 and a 60%-fold increase in fluorescent intensity (Figure 4d).

### 3.5. Effect of BV6 Cotreated with TRAIL and TNFα on MCF7 and MDA-MB-231 Cell Viability, Cell Morphology and Apoptosis

**Figure 5:**
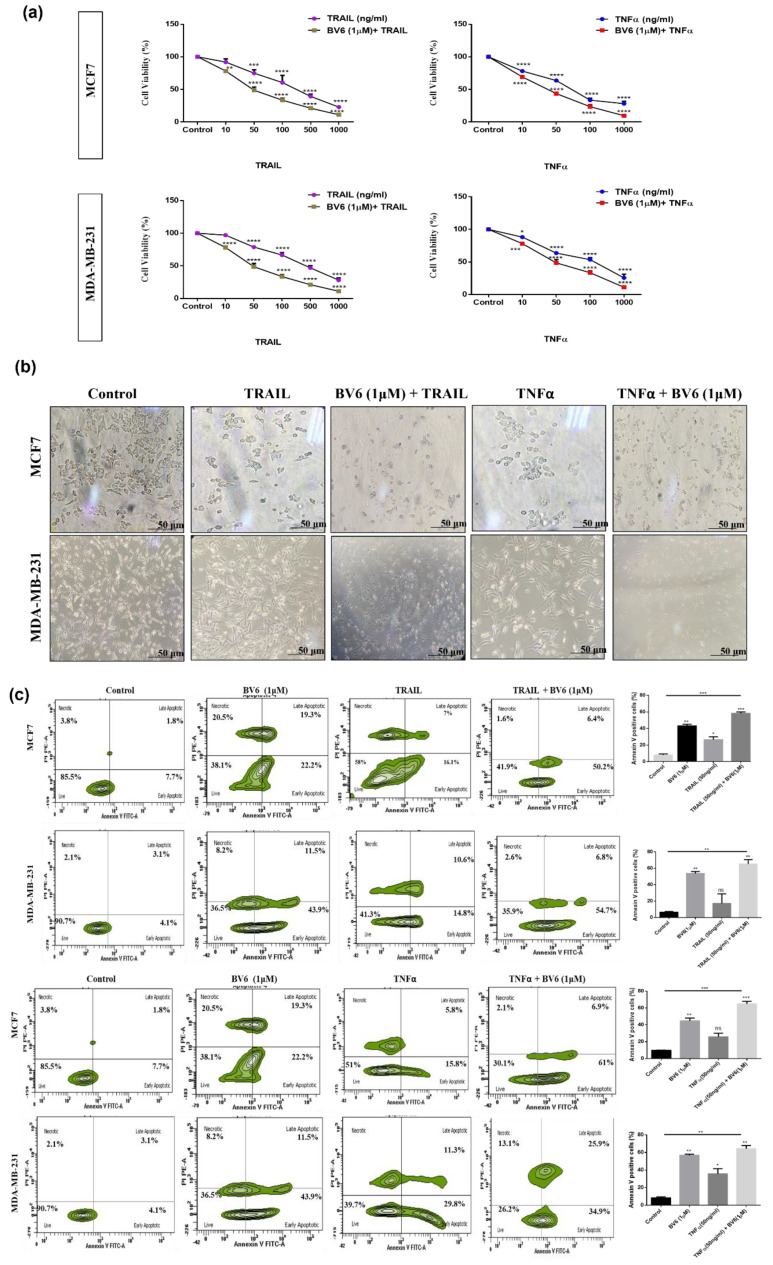
(**a**) Effect of TRAIL, TNFα and their combination with BV6 on MCF7 and MDA-MB-231 cells by MTT assay: Treatment of cancer cells was done with an increasing concentration of TRAIL, TNFα alone and in combination with 1 μM of BV6 for 24 h. (**b**) Effect of TRAIL (50 ng/mL), TNFα (50 ng/mL) and their combination with BV6 (1 μM) on MCF7 and MDA-MB-231 cells morphology: cells with no treatment showed well adhered normal morphology whereas, after treatment, cells displayed shrunk, round and suspended in culture medium; scale bar 50 μm. (**c**) Apoptosis analysis by flow cytometry; Quadrants showing the proportion of live cells, early apoptotic cells, late apoptotic cells and necrosis in control and TRAIL (50 ng/mL), TNFα (50 ng/mL) and their combination with BV6 (1 μM) treated cells. Histogram profile demonstrating the percentage of Annexin V positive cells, i.e., apoptosis-induced. The data represented is statistically significant: (*), (**), (***), and (****) show *p* < 0.05, *p* < 0.01, *p* < 0.001, and *p* < 0.0001, respectively, and is carried out in triplicates displayed as mean ± SEM.

MTT assay was performed with different concentrations of TRAIL and TNFα treatment alone and in combination with BV6 (1 μM) to investigate its antitumor activity and cell viability on the breast cancer cells MCF7 and MDA-MB-231. After 24 h, results indicated that individual treatment with both TRAIL and TNFα inhibits the cell viability in a dose-dependent manner. However, BV6 combination with TRAIL and TNFα strongly inhibited the growth and survival of both breast cancer cell lines. (Figure 5a). Co-treatment of BV6 with TRAIL and TNFα reduced cell viability to a greater extent than either alone.

An inverted phase-contrast microscopy study showed that cells become round, shrunk, irregular and suspended in culture media with the TRAIL and TNFα treatment. Whereas BV6, in synergy with TRAIL and TNFα treatment, worsens the morphology of both breast cancer cells. This indicates that in response to BV6 combination with TRAIL and TNFα, MCF7 and MDA-MB-231 cells shrink and detach from the surface, along with a decline in the number of cells (Figure 5b).

Apoptosis induction after TRAIL and TNFα treatment alone and in combination with BV6 (1 μM) was analyzed using Annexin V/PI flow cytometry. In MCF7, a 30–45% increase of apoptosis was observed in BV6 in combination with TRAIL and TNFα treatment compared to individual treatment with TRAIL and TNFα, i.e., 20–25%. Whereas, in MDA-MB-231 breast cancer cells, an increase in apoptosis is observed from 20–30% in BV6 combined with TRAIL and TNFα compared to individual treatment with TRAIL and TNFα, i.e., 25–41% (Figure 5c).

### 3.6. Autophagy in MCF7 and MDA-MB-231 Cells Treated with TRAIL, TNFα and in Synergy with BV6

**Figure 6:**
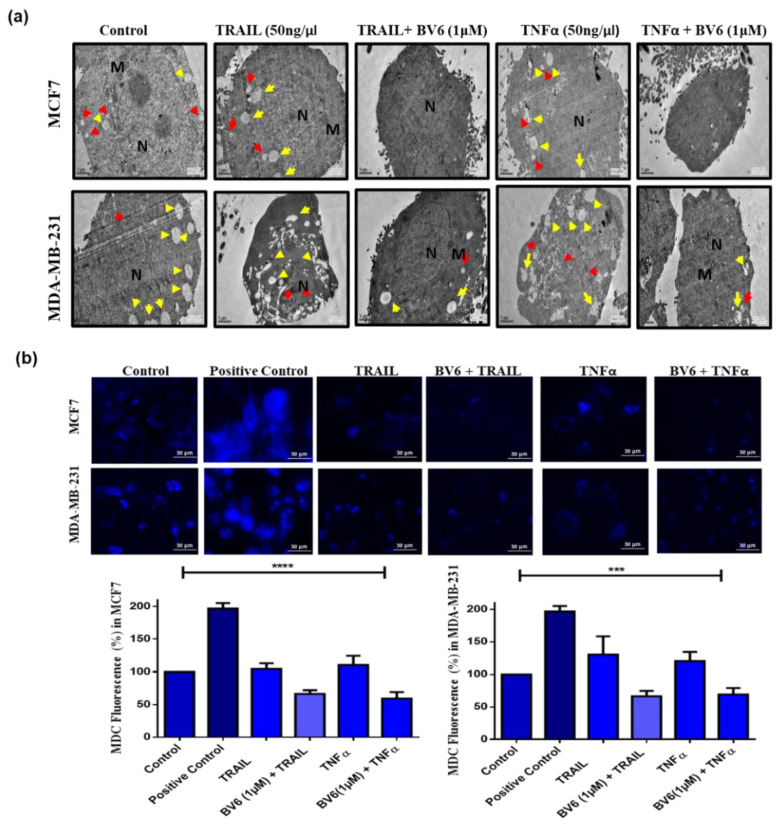
Autophagy in MCF7 and MDA-MB-231 cells treated with TRAIL (50 ng/mL), TNFα (50 ng/mL) and their combination with BV6 (1 μM): (**a**) TEM images show a normal double-membrane nucleus surrounded with normal-appearing mitochondria along with accumulated autophagic vacuoles such as autophagosomes and autolysosomes. M represents mitochondria, N represents Nucleus (**b**) Fluorescence images captured from fluorescence microscopy of MDC staining showing stained autophagic vacuoles or autophagosome accumulation in untreated (control), starved cells (Positive control) and treated cells. Histogram profile representing the mean fluorescence intensity of TRAIL (50 ng/mL), TNFα (50 ng/mL) and their combination with BV6 (1 μM) treated MCF7 and MDA-MB-231 compared to the fluorescent intensity of untreated control and positive control. Data represented is statistically significant: (***), and (****) show *p* < 0.001, and *p* < 0.0001, respectively, and is carried out in triplicates displayed as mean ± SEM.

Transmission Electron Microscopy (TEM) displayed an increase in the number of autophagosomes and autolysosomes (red and yellow arrows, respectively) in TRAIL and TNFα treatment after 24 h. At the same time, in treatment with BV6 in synergy with TRAIL and TNFα, the number of autophagic vacuoles decreased compared to the control and TRAIL and TNFα treatment individually (Figure 6a). It is suggested that BV6, in combination with TRAIL and TNFα, inhibits the autophagy process, whereas TRAIL and TNFα treatment increases autophagy in MCF7 and MDA-MB-231 breast cancer cells. Fluorescent images displayed MDC dye-stained autophagosomes in cells. The histogram profile shows a 30–40% and 30% decrease in mean fluorescence intensity in combinational treatment of BV6 with TRAIL and TNFα in MCF7 and MDA-MB-231 cell lines, respectively, compared to the fluorescent intensity of untreated control of both cell lines. At the same time, individual treatment of TRAIL and TNFα in MCF7 and MDA-MB-231 cell lines displayed an increase in 10–50% of fluorescent intensity in both breast cancer cell lines. In comparison to the control group, starved cells show about a 96%-fold increase in MCF7 and a 60%-fold increase in fluorescent intensity (Figure 6b).

**Figure 7:**
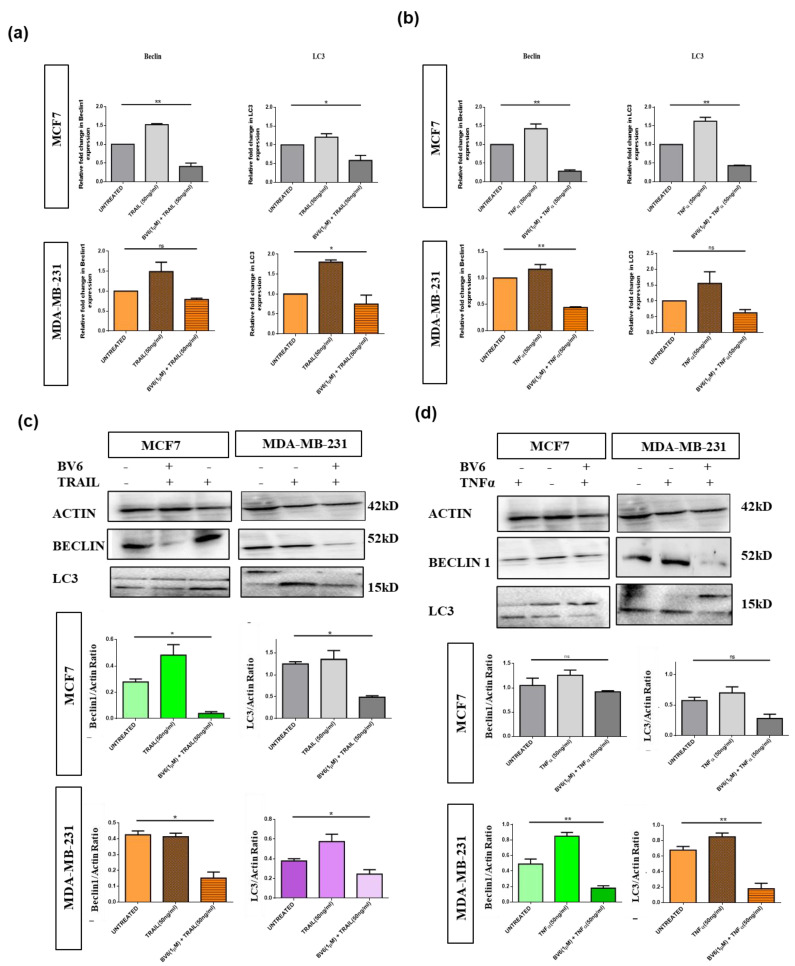
Expression analysis of autophagy biomarkers (Beclin1 and LC3) in MCF7 and MDA-MB-231 cells treated with TRAIL (50 ng/mL), TNFα (50 ng/mL) and their combination with BV6 (1 μM) with the help of real-time PCR and western blotting: (**a**) The relative fold change in Beclin-1 and LC3 mRNA expression levels normalized by Actin in both breast cancer cell lines treated with TRAIL (50 ng/mL) alone and in synergy with BV6 (1 μM). (**b**) The relative fold change in the Beclin-1 and LC3 mRNA expression levels normalized by Actin in both breast cancer cell lines treated with TNFα (50 ng/mL) alone and in synergy with BV6 (1 μM). (**c**) Western Blot images showing the protein bands of untreated control and treated breast cancer cells with TRAIL (50 ng/mL) alone and in synergy with BV6 (1 μM). Histogram displaying the Beclin-1 and LC3 protein expression levels normalized by Actin. (**d**) Western Blot images showing the protein bands of untreated control and treated breast cancer cells with TNFα (50 ng/mL) alone and in synergy with BV6 (1 μM). Histogram displaying the Beclin-1 and LC3 protein expression levels normalized by Actin The experiments are carried out in triplicates displayed as mean ± SEM, and the data represented is statistically significant: (*) and (**) show *p* < 0.05 and *p* < 0.01, respectively.

Expression of autophagic markers, i.e., Beclin1 and LC3, was evaluated with the help of real-time PCR and western blotting. Downregulation of beclin1 and LC3 mRNA and protein expression is observed in response to the BV6 in combination with TRAIL and TNFα in both MCF7 and MDA-MB-231 breast cancer cell lines compared to the mRNA and protein expressions of biomarkers in the untreated group and individual treatments with TRAIL and TNFα. Lone treatment of TRAIL and TNFα increases the mRNA and protein expression of beclin1 and LC3 in both breast cancer cell lines (Figure 7a–d).

## 4. Discussion

Despite having several cancer treatment regimes availabilities, still, breast cancer is the most frequently diagnosed cause of morbidity among women globally. The ability of cancer cells to escape from cell death mechanisms makes them resistant to therapies. IAPs and autophagy are found to play a substantial role in the resistance to conventional therapies and the reoccurrence of cancer [4,19,20]. However, in normal cells, IAPs are highly regulated to avoid unwanted apoptosis. It is inhibited by the natural SMAC protein system, which plays a critical role in inhibiting apoptosis by blocking caspase proteins [2,4,5]. There are eight mammalian IAPs, and among them, XIAP, cIAP1, cIAP2, and Survivin have major anti-apoptotic roles. Overexpression of these IAPs promotes and maintains carcinogenesis and tumor spread [21]. At the clinical level, 99% of XIAP expression was reported in breast cancer patients. The significantly higher expression of XIAP and survivin was in advanced tumors, demonstrating the most noteworthy prognostic importance in breast cancer [7]. Target inhibition of these IAPs mitigates therapeutic resistance in breast cancer [9,22,23].

Upon apoptotic stimuli, along with cytochrome c from the mitochondria, a minute amount of SMAC proteins is also released into the cytosol to inhibit IAPs. Some evidence is largely attributed to the disruption of SMAC released in carcinogenesis. In a clinical study, longer overall survival and significant tumor remission rate in cancer patients were observed with higher expression levels of SMAC [6]. Therefore, novel drugs were developed, and they are known as “SMAC mimetics”, which mimic like second mitochondrial-derived activator of caspases (SMAC) proteins. Earlier studies show that inhibiting the expression of IAPs by SMAC mimetics helps cancer cells undergo apoptosis and makes cancer cells sensitive to therapies [10,24,25]. Various SMAC mimetic compounds are synthesized and are effective against cancer, and most of them are under pre-clinical and clinical trials [2,6,8]. In an animal model, it has been observed that the early-stage metastasis of MDA-MB-231 cells from bone to the lung was prevented effectively after SMAC mimetic (SM-164) administration [26]. Inflammatory breast cancer is one of the most lethal subtypes of breast cancer shows that Smac mimetic Birinapant induces apoptosis and enhances TRAIL potency in cells in an IAP-dependent mechanism [27]. In a study, BV6 causes the induction of apoptosis via IAP degradation and sensitization by producing death ligands, tumor necrosis factor-α (TNF-α), and TNF-related apoptosis-inducing ligands (TRAILs) in various human cancer cell lines [11].

In the present study, smac mimetic BV6 affects the viability of MCF7 and MDA-MB-231 cells in a concentration-dependent manner. The SMAC mimetics treatments in breast cancer cells showed characteristic features of apoptosis, such as a change in cell morphology, distorted nuclear structure and cell detachment. Furthermore, induction of apoptosis was validated by different staining experiments, such as Acridine Orange/Ethidium Bromide and Annexin V/PI staining. SMAC mimetic compound BV6 induces apoptosis significantly via inhibiting IAPs, i.e., XIAP, cIAP1, cIAP2 and survivin, demonstrated by the IAP’s mRNA and protein expression analysis. In addition, treatment of BV6 demonstrates a decrease in cell migration/invasiveness, which contradicts the result obtained by El-Mesery and colleagues [11]. It suggests that these compounds can remove or decrease therapy resistance and may prevent the metastasis of cancer cells.

Autophagy is a conserved, stress-adaptive process that aids cancer cells in escaping from cell death. It provides nutrients to rapidly dividing cancer cells and develops resistance against therapies. Thus, acting as a tumor progressor at the advanced stage of cancer. However, at an early stage of carcinogenesis, autophagy displayed a tumor suppressor role via degrading damaged macromolecules, DNA, organelles etc. Moreover, defect in this programmed cell death type II can cause cancer as well as helps in overcoming the resistance via causing the death of therapies-resistant cancer cells [14,15,16,28,29].

For the first time, our study discloses the effect of smac mimetic compound BV6 on autophagy in MCF7 and MDA-MB-231 breast cancer cell lines. LC3I to LC3II conversion contributes to the formation of autophagosomes, and Beclin1 is necessary for the induction/initiation of autophagy or the formation of autophagosomes. Therefore, LC3II and Beclin have usually considered markers of autophagy [16,30,31,32]. Evidence supports the autophagic pro-survival effect in various cancers, and autophagy inhibition helps overcome therapeutic resistance via apoptosis induction [33,34,35,36]. IAPs family members with a negative modulating effect on apoptosis may also be able to modulate autophagy since various components or molecules of autophagy and apoptosis pathways cross-inhibit or stimulate each other [16,37]. APG-1387, a smac mimetic, targets cIAP1, cIAP2, and XIAP, inducing autophagy and cell death in human ovarian cancer cells [38]. Another IAP antagonist, YM155, causes the death of breast cancer cells in an autophagy-dependent manner [39]. Similarly, GDC-0152 induces autophagy and promotes apoptosis in HL-60 cells [40]. On the contrary, autophagosome and lysosome fusion in mouse embryonic cells (MEFs) was inhibited by smac mimetic LCL161 [41,42]. In another study, Beclin1 expression is positively regulated by XIAP and cIAP1, which is crucial for the biogenesis of autophagosome, suggesting that IAP, especially XIAP, can act as an autophagy suppressor [43]. Thus, in different cells under different circumstances, IAPs seem to display differential autophagic roles.

In the present study, IAP antagonist BV6 downregulates autophagy. The mRNA and protein expression analysis of autophagy markers, i.e., Beclin1 and LC3, using real-time PCR and western blotting demonstrated the downregulation of Beclin1 and LC3 expression in BV6 treated MCF7 and MDA-MB-231. This is further substantiated by transmission electron microscopy (TEM); qualitative analysis demonstrated a decrease in autophagic vacuoles (autophagosomes/autolysosomes) accumulation in BV6 treated group compared to untreated cells. Moreover, we also found that the cotreatment of BV6 with TRAIL and TNFα enhances the anti-tumor properties compared to the lone treatment of TRAIL and TNFα. SMAC mimetic BV6 induces cell death, inhibits autophagy, and sensitizes breast cancer cell lines to TNF-α and TRAIL-induced apoptosis. In various in-vivo and in-vitro studies of cancer such as breast cancer, colon cancer etc., autophagy was found to protect the cancer cells from the effect of these death ligands [44,45]. Therapeutic targeting of autophagosome formation could be a novel molecular avenue to reduce the resistance of TRAIL in breast cancer. However, more studies, including in-vivo models, need to be done for further validation and to strengthen the conclusion drawn from this study.

## 5. Conclusions

The study concludes that the SMAC compound (BV6) shows an anticancer effect on both MCF-7 and MDA-MB-231 breast cancer cell lines by targeting IAPs and autophagic proteins. SMAC mimetic compound BV6 downregulates IAPs (XIAP, cIAP1, cIAP2, Survivin) and induces apoptosis. For the first time, we studied the effect of SMAC mimetic compound on type II cell death mechanism, i.e., Autophagy. We found that BV6 treatment downregulates autophagy. The autophagy analysis concludes that SMAC mimetic BV6 decreases the factors which are involved in developing resistance to various anticancer conventional therapies. i.e., Inhibitors of Apoptosis (IAPs) and autophagy. In addition, we also found that cotreatment of BV6 with TRAIL and TNFα downregulates autophagy which could serve as a mechanism for the sidestepping of TRAIL/TNFα resistance in tumor cells with different apoptosis defects. Thus, suggesting that BV6 could be used unaided or in combination with anticancer therapies/drugs to overcome the problem of cancer reoccurrence and resistance. Besides, IAPs and autophagic proteins can act as potential target molecules for the development of novel anti-cancer drugs or smac mimetic drugs. The utilization of smac mimetic compounds could be employed as a therapeutic approach to eliminate malignant cells with a high apoptotic threshold or dysfunction apoptosis in cancerous cells.

## Data Availability

Not applicable.
